# CCDC88A, a prognostic factor for human pancreatic cancers, promotes the motility and invasiveness of pancreatic cancer cells

**DOI:** 10.1186/s13046-016-0466-0

**Published:** 2016-12-05

**Authors:** Aki Tanouchi, Keisuke Taniuchi, Mutsuo Furihata, Seiji Naganuma, Ken Dabanaka, Masashi Kimura, Ryohei Watanabe, Takuhiro Kohsaki, Takahiro Shimizu, Motoaki Saito, Kazuhiro Hanazaki, Toshiji Saibara

**Affiliations:** 1 Department of Gastroenterology and Hepatology; Kochi Medical School, Kochi University, Kochi, Japan; 2 Departments of Endoscopic Diagnostics and Therapeutics, Kochi Medical School, Kochi University, Kohasu, Oko-cho, Nankoku, Kochi, 783-8505 Japan; 3 Department of Pathology, Kochi Medical School, Kochi University, Kochi, Japan; 4 Department of Surgery, Kochi Medical School, Kochi University, Kochi, Japan; 5 Department of Surgery, Matsuyama Shimin Hospital, Matsuyama, Japan; 6 Department of Pharmacology, Kochi Medical School, Kochi University, Kochi, Japan

**Keywords:** Akt-binding protein, Pancreatic cancer, Cell invasion, Cell protrusions, AMPK1

## Abstract

**Background:**

Coiled-Coil Domain Containing 88A (CCDC88A) was identified as a substrate of the serine/threonine kinase Akt that is capable of binding to the actin cytoskeleton. The aim of this study was to investigate the potential role of CCDC88A in the migration and invasiveness of pancreatic ductal adenocarcinoma (PDAC) cells.

**Methods:**

Immunohistochemistry was performed to determine whether high CCDC88A expression in human PDAC tissues is correlated with poor prognosis. Immunoprecipitation, immunoblotting and immunocytochemistry were performed to determine the intracellular distribution of CCDC88A, and its association with the serine/threonine kinase Akt and actin-filaments in PDAC cells. Phosphoprotein array analysis was performed to determine CCDC88A-associated intracellular signaling pathways. Finally, immunofluorescence analyses and Matrigel invasion assays were performed to examine the effects of CCDC88A on the formation of cell protrusions and PDAC cell invasion.

**Results:**

Expression of CCDC88A in PDAC tissue was significantly correlated with overall survival. CCDC88A was co-localized with peripheral actin structures in cell protrusions of migrating PDAC cells. Knockdown of CCDC88A inhibited the migration and invasiveness of PDAC cells through a decrease in cell protrusions. Although CCDC88A has been previously reported to be a binding partner and substrate of Akt, the level of active Akt was not associated with the translocation of CCDC88A towards cell protrusions. CCDC88A-dependent promotion of cell migration and invasiveness was not modulated by Akt signaling. Knockdown of CCDC88A decreased phosphorylated Src and ERK1/2 and increased phosphorylated AMPK1 in PDAC cells. Knockdown of AMPK1 inhibited the migration and invasiveness of PDAC cells. The combined data suggest that CCDC88A may be a useful marker for predicting the outcome of patients with PDAC and that CCDC88A can promote PDAC cell migration and invasion through a signaling pathway that involves phosphorylation of Src and ERK1/2 and/or dephosphorylation of AMPK1.

**Conclusions:**

CCDC88A was accumulated in cell protrusions, contributed to the formation of membrane protrusions, and increased the migration and invasiveness of PDAC cells.

**Electronic supplementary material:**

The online version of this article (doi:10.1186/s13046-016-0466-0) contains supplementary material, which is available to authorized users.

## Background

Coiled-Coil Domain Containing 88A (CCDC88A), also termed Girdin, was identified as a novel substrate of the serine/threonine kinase Akt (also called protein kinase B) that is capable of binding to the actin cytoskeleton [1]. Thus, CCDC88A is a novel component of the phosphatidylinositol 3-kinase (PI3-K)⁄Akt signaling pathway, which is a core-signaling transduction pathway in cancer [2]. Akt regulates the invasiveness and metastasis of fibrosarcoma cells in a manner that is highly dependent on its kinase activity and its membrane-translocating ability [3]. The COOH terminal domain of CCDC88A contains its Akt- and actin-binding sites and is also assumed to contribute to the interaction of CCDC88A with the plasma membrane [4]. Additionally, CCDC88A can form dimers through the NH_2_ terminal domain, indicating that it may function as an actin-cross-linking protein [4]. Upon stimulation of cells with various types of growth factors, Akt phosphorylates CCDC88A at Ser-1416 in the COOH terminal domain, which plays an important role in remodeling of the actin cytoskeleton during cell migration in fibroblasts [1] and in colorectal cancer [5]. The phosphorylation of CCDC88A by Akt occurs at the leading edge, which is required for directional cell migration and which, in the case of cancer cells, ultimately leads to invasion and metastasis [6]. Additionally, CCDC88A is phosphorylated by active Akt following stimulation of breast cancer cells with insulin-like growth factor (IGF-I), and this phosphorylation plays an important role in IGF-I-dependent cell movement [7]. Furthermore, CCDC88A-mediated Akt activation is positively regulated by Gαi, a Gα subunit of heterotrimeric G proteins that act as intracellular transducers to mediate signals from G protein-coupled receptors (GPCR) [8, 9]. The guanine nucleotide exchange motif located in the COOH terminal domain of CCDC88A activates and sequesters Gαi, thereby enhancing Akt signaling [9]. Thus, CCDC88A serves as a mediator of the GPCR-Akt signaling pathway.

Pancreatic ductal adenocarcinoma (PDAC) is among the deadliest of cancers because PDAC cells are highly invasive and easily invade surrounding tissues, and they metastasize at an early stage [10]. We recently reported that an RNA-binding protein, insulin-like growth factor-2 mRNA-binding protein 3 (IGF2BP3), and IGF2BP3-bound mRNAs, are accumulated in cell protrusions of PDAC cells [11]. IGF2BP3-bound mRNAs such as ADP-ribosylation factor 6 (*ARF6*) and Rho guanine nucleotide exchange factor 4 (*ARHGEF4*) are subsequently translated in membrane protrusions; in turn, these locally translated proteins influence the formation of additional membrane protrusions and thereby increase the motility and invasiveness of the PDAC cells [11, 12]. IGF2BP3 was also found to bind to *CCDC88A* mRNA [11]. These findings indicate that local protein expression of CCDC88A in cell protrusions may modulate the motility and invasiveness of PDAC cells.

In this study, we analyzed the expression levels of CCDC88A in human PDAC tissues by using immunohistochemistry and evaluated whether high CCDC88A expression is correlated with poor prognosis. To determine whether CCDC88A expression might play a crucial role in the outcome of PDAC through modulation of the migration and invasiveness of cancer cells, or through its association with Akt, we next evaluated the role of CCDC88A in the control of PDAC cell migration and invasion. In contrast to some previous reports, knockdown of CCDC88A did not alter the intracellular distribution of Akt in PDAC cells, and CCDC88A promoted cell migration and invasiveness in an Akt-independent manner.

## Results

### CCDC88A expression in human PDAC tissues

**Fig. 1:**
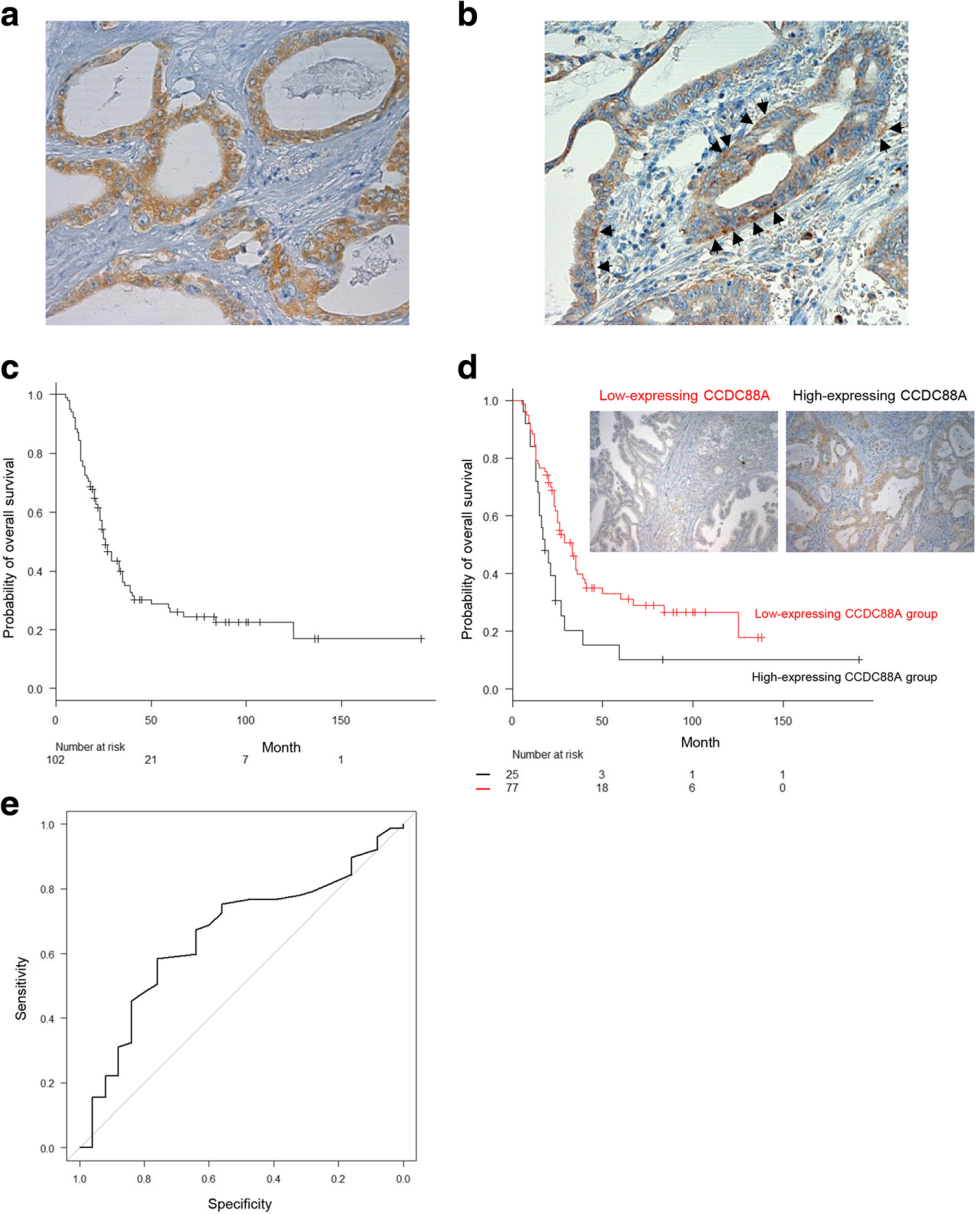
Association of high expression of CCDC88A with a poor outcome in PDAC patients. **a**, **b**. Immunohistochemical staining of PDAC tissues using anti-CCDC88A antibody. CCDC88A staining was present in the cytoplasm (**a**) and in the basolateral portions (**b**) of tumor cells. Arrows, CCDC88A localized basolaterally. Magnifications: ×200. **c**, **d**. Kaplan-Meier analysis of (**c**) PDAC-specific survival and (**d**) overall survival according to CCDC88A expression. **e**. ROC curve of 102 PDAC cases for analysis of the impact of the total immunohistochemical score of CCDC88A on prognosis

We examined CCDC88A expression in surgical specimens from 102 patients with PDAC by immunohistochemical analysis. A Histoscore scoring method [13], which takes into account both the extent of expression and the staining intensity of CCDC88A, was employed. Expression levels of CCDC88A were evaluable in all 102 cases, and these cases were classified into low-expressing (75.5%, *n* = 77, total immunohistochemical score = 2 and 3) and high-expressing (24.5%, *n* = 25, total immunohistochemical score = 4, 5 and 6) CCDC88A groups (Table 1). CCDC88A mostly localized in the cytoplasm of cell bodies (Fig. 1a). Among the high-expressing CCDC88A group, seven PDAC cases showed CCDC88A immuno-reactivity that was restricted to the basolateral portion of the cell in more than 50% of PDAC tumor cells (Fig. 1b). Weaker staining of CCDC88A in the basolateral portions of cells in less than 50% of PDAC tumor cells was seen in all of the low-expressing CCDC88A PDAC cases (data not shown). Pancreatic ducts were not obviously stained in normal pancreas, and normal brain, lung, liver and kidney were not obviously stained with the CCDC88A antibody (data not shown).

**Table 1:** Summary of characteristics in 102 patients of pancreatic cancer

Caracteristics	Percentage (%)	
Age at surgery		
40–50	3.9	[*n* = 4]
50–60	16.7	[*n* = 17]
60–70	31.4	[*n* = 32]
70–80	40.2	[*n* = 41]
>80	7.8	[*n* = 8]
Gender		
Male	54.9	[*n* = 56]
Female	45.1	[*n* = 46]
Stage^a^		
0	2.0	[*n* = 2]
IA	4.0	[*n* = 4]
IB	7.8	[*n* = 8]
IIA	31.4	[*n* = 32]
IIB	47.9	[*n* = 49]
III	2.9	[*n* = 3]
IV	4.0	[*n* = 4]
Primary tumor^a^		
Tis	2.0	[*n* = 2]
T1	6.0	[*n* = 6]
T2	14.5	[*n* = 15]
T3	75.5	[*n* = 77]
T4	2.0	[*n* = 2]
Regional lymph nodes^a^		
N0	46.1	[*n* = 47]
N1	53.9	[*n* = 55]
M0	96.1	[*n* = 98]
M1	3.9	[*n* = 4]
Histology^b^		
PanIN	2.0	[*n* = 2]
well	30.4	[*n* = 31]
moderate	55.8	[*n* = 57]
poor	11.8	[*n* = 12]
Venous invasion^b^		
v0	55.4	[*n* = 57]
v1	30.7	[*n* = 31]
v2	10.9	[*n* = 11]
v3	3.0	[*n* = 3]
Lymphatic invasion^b^		
ly0	42.6	[*n* = 43]
ly1	33.6	[*n* = 34]
ly2	19.9	[*n* = 21]
ly3	3.9	[*n* = 4]
CCDC88A expression		
Low	75.5	[*n* = 77]
High	24.5	[*n* = 25]

^a^Classified according to the classification of International Union against Cancer

^b^Classified according to the classification of pancreatic cancer of Japan Pancreas Society; PanIN, pancreatic intraepithelial neoplasia

### Association between CCDC88A expression, clinicopathological characteristics and overall survival

We next analyzed the relationship between CCDC88A expression and clinicopathological features as shown in Table 2. There was no significant association between CCDC88A expression and the clinicopathological features examined. In addition, there was no significant correlation between high-expressing CCDC88A PDAC cases that exhibited CCDC88A expression in the basolateral portions of more than 50% of the PDAC tumor cells and any of the clinicopathological features (data not shown).

**Table 2:** Correlation between CCDC88A expression and clinicopathological parameters

	CCDC88A expression				*P*
	Low		High		
Stage^a^	percentage (%)				0.938
0	2.6	[*n* = 2]	0	[*n* = 0]	
IA	3.9	[*n* = 3]	4.0	[*n* = 1]	
IB	7.8	[*n* = 6]	8.0	[*n* = 2]	
IIA	29.9	[*n* = 23]	36.0	[*n* = 9]	
IIB	50.6	[*n* = 39]	44.0	[*n* = 11]	
III	1.3	[*n* = 1]	4.0	[*n* = 1]	
IV	3.9	[*n* = 3]	4.0	[*n* = 1]	
Primary tumor^a^					0.656
Tis	2.6	[*n* = 2]	0	[*n* = 0]	
T1	6.5	[*n* = 5]	4.0	[*n* = 1]	
T2	13.0	[*n* = 10]	20.0	[*n* = 5]	
T3	76.6	[*n* = 59]	72.0	[*n* = 18]	
T4	1.3	[*n* = 1]	4.0	[*n* = 1]	
Regional lymph nodes^a^					0.644
N0	44.2	[*n* = 34]	52.0	[*n* = 13]	
N1	55.8	[*n* = 43]	48.0	[*n* = 12]	
Distant metastasis^a^					1
M0	96.1	[*n* = 74]	96.0	[*n* = 24]	
M1	3.9	[*n* = 3]	4.0	[*n* = 1]	
Histology^b^					0.9391
PanIN	2.6	[*n* = 2]	0	[*n* = 0]	
Well	29.9	[*n* = 23]	32.0	[*n* = 8]	
Moderate	54.5	[*n* = 42]	60.0	[*n* = 15]	
Poor	13.0	[*n* = 10]	8.0	[*n* = 2]	
Venous invasion^b^					0.741
v0 + v1	87	[*n* = 67]	84.0	[*n* = 21]	
V2 + v3	13	[*n* = 10]	16.0	[*n* = 4]	
Lymphatic invasion^b^					0.789
ly0 + ly1	76.6	[*n* = 59]	72.0	[*n* = 18]	
ly2 + ly3	23.4	[*n* = 18]	28.0	[*n* = 7]	
Recurrence					0.133
Yes	67.5	[*n* = 52]	84.0	[*n* = 21]	
No	22.5	[*n* = 25]	16.0	[*n* = 4]	

^a^Classified according to the classification of International Union against Cancer

^b^Classified according to the classification of pancreatic cancer of Japan Pancreas Society; PanIN, pancreatic intraepithelial neoplasia

The follow-up period for survivors of 102 patients with PDAC ranged from 18 to 192 months (median, 64.0 mo). The median survival time was 26 months (95% confidence interval [CI], 23–33); the 3 year survival rate was 35.1% (95% CI, 25.6–44.8); and the 5 year survival rate was 25.9% (95% CI, 17.2–35.5). Kaplan-Meier plots showed that there was a significant difference in overall survival rates (*P* = 0.012) between groups with high and low CCDC88A expression (Fig. 1c, d). Univariate and multivariate analyses were used to assess the prognostic value of CCDC88A expression in PDAC. Stage III and IV (the Union for International Cancer Control (UICC) TNM classification) (hazard ratio [HR], 3.035; 95% CI, 1.301–7.081; *P* = 0.010) and high CCDC88A expression (HR, 0.497; 95% CI, 0.295–0.836; *P* = 0.008) were independent and significant prognostic factors for worse patient survival by univariate Cox regression analysis (Table 3). Multivariate survival analysis indicated that stage III and IV (HR, 11.10; 95% CI, 3.012–40.92; *P* < 0.001) and high CCDC88A expression (HR, 0.519; 95% CI, 0.306–0.877; *P* = 0.014) proved to be independent prognostic factors for worse patient survival (Table 3). The effects of stage III and IV, and CCDC88A expression on overall survival were similar between univariate and multivariate analyses. Furthermore, the overall survival rate of the high-expressing CCDC88A group was compared with that of the low-expressing CCDC88A group using a receiver operating characteristic (ROC) curve (Fig. 1e). Such a ROC-curve may be useful for additional application in survival analysis [14]. The area under the curve (AUC) of the ROC curve was 0.657 (95% CI, 0.536–0.779). The median survival time, 3 year survival rate and 5 year survival rate of the low-expressing CCDC88A group were 33 months (95% CI, 24–39), 39.8% (95% CI, 28.4–51.0) and 31.1% (95% CI, 20.3–42.4), respectively. The median survival time, 3 year survival rate and 5 year survival rate of the high-expressing CCDC88A group were 18 months (95% CI, 14–24), 20.4% (95% CI, 6.8–38.8) and 10.2% (95% CI, 1.8–27.2), respectively.

**Table 3:** Univariate and multivariate analysis of prognostic factors for overall survival

	Overall survival			
	Univariate		Multivariate	
	HR (95% CI)	*P*	HR (95% CI)	*P*
Stage^a^				
0 + IA + IB IIA IIB III + IV	1.0 (reference) 1.159 (0.714–1.881) 1.356 (0.854–2.151) 3.035 (1.301–7.081)	0.549 0.196 0.010	1.0 (reference) 5.705 (1.929–16.88) 6.270 (2.160–18.20) 11.10 (3.012–40.92)	1.650e–03 7.361e–04 2.985e–04
Age	1.025 (0.998–1.053)	0.067	1.024 (0.996–1.052)	0.086
Gender	1.249 (0.771–2.023)	0.366	1.236 (0.759–2.012)	0.393
CCDC88A expression	0.497 (0.295–0.836)	0.008	0.519 (0.306–0.877)	0.014
Diameter of primary tumor	1.338 (1.176–1.524)	1.045e–05		
Histology^b^	1.383 (0.846–2.261)	0.1965		
Lymphatic invasion^b^ (ly0 + ly1 or ly2 + ly3)	1.269 (0.751–2.145)	0.3733		
Venous invasion^b^ (v0 + v1 or v2 + v3)	1.928 (1.034–3.593)	0.0388		
Intrapancreatic nerve invasion^b^ (n0 + n1 or n2 + n3)	1.500 (0.947–2.377)	0.0839		

^a^Classified according to the classification of International Union against Cancer

^b^Classified according to the classification of pancreatic cancer of Japan Pancreas Society

### Subcellular localization of CCDC88A in fibronectin-stimulated PDAC cells

**Fig. 2:**
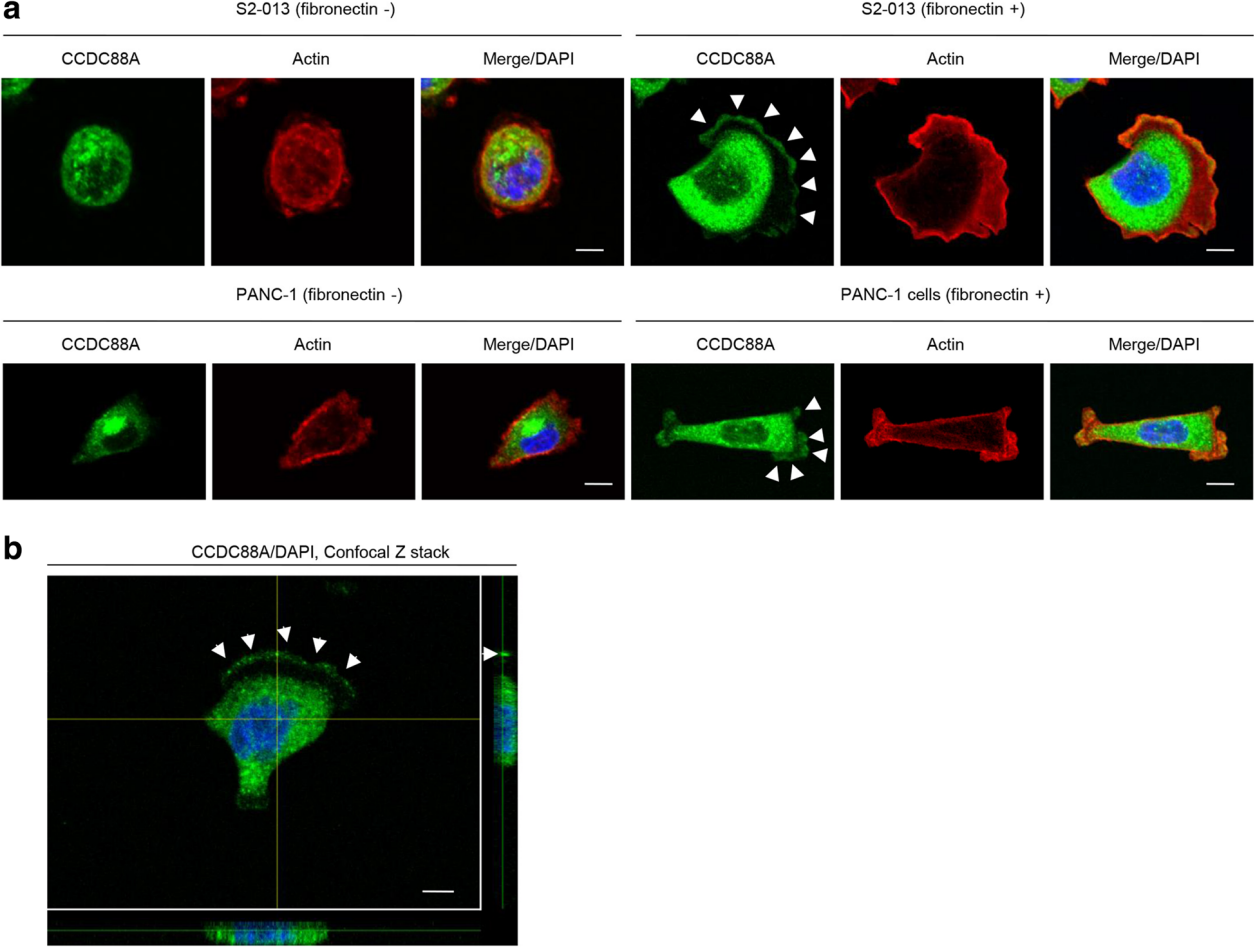
Subcellular localization of CCDC88A in PDAC cells. **a**. Confocal immunofluorescence microscopic images of S2-013 and PANC-1 cells that were cultured with or without fibronectin and were then labeled with anti-CCDC88A antibody (*green*) and phalloidin (*red; actin filaments*). Arrows, CCDC88A localized in cell protrusions. Blue, nuclear DAPI staining. Bars, 10 μm. **b**. Confocal Z stack images show nuclear DAPI staining (*blue*), abundant cytoplasmic CCDC88A and the accumulation of CCDC88A (*green*) in membrane protrusions of fibronectin-stimulated S2-013 cells. Arrows, CCDC88A localized in cell protrusions. The lower and right panels in the confocal Z stack show a vertical cross-section (*yellow lines*) through the cells. Bar, 10 μm

We performed immunocytochemistry to determine the subcellular localization of CCDC88A in two types of cultured PDAC cells, moderately differentiated (S2-013 cell line) and poorly differentiated (PANC-1 cell line) PDAC cells. It has previously been shown that, when S2-013 cells that were initially in suspension attach to an immobilized fibronectin substrate, nascent membrane protrusions (de novo formation of actin patches at the cell periphery) form, and, as these protrusions mature, they promote cell motility [15, 16]. There were fewer membrane protrusions formed by S2-013 and Panc-1 cells when the cells were cultured without fibronectin than when the cells were grown on fibronectin [17]. Our analysis of fibronectin-stimulated S2-013 and PANC-1 cells indicated that CCDC88A was mainly present in the cytoplasm of the cell bodies. It should be noted that there was a greater accumulation of CCDC88A in membrane protrusions that contained many peripheral actin structures in S2-013 and PANC-1 cells that were cultured on fibronectin, than in the corresponding cells cultured without fibronectin (Fig. 2a). Z stack panels showed that fibronectin-stimulated S2-013 cells exhibited intracellular expression of CCDC88A in membrane protrusions (Fig. 2b).

### Effects of knockdown of CCDC88A on cell migration and invasion of PDAC cells

**Fig. 3:**
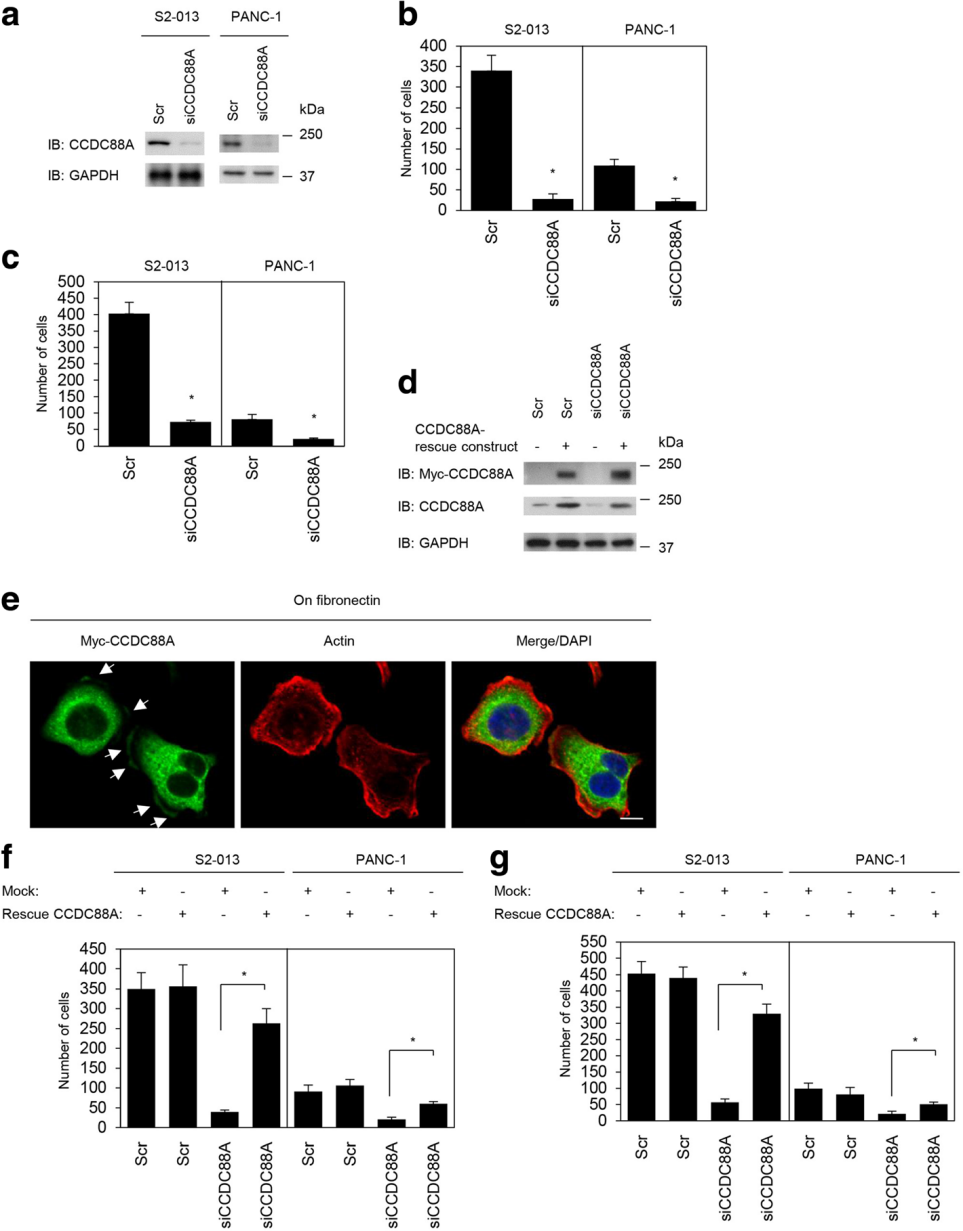
Roles of CCDC88A in cell migration and invasion. **a**. Western blotting of CCDC88A following transient transfection of S2-013 and PANC-1 cells with a single mixture containing four different siRNA oligonucleotides targeting *CCDC88A* (siCCDC88A) or negative scrambled control (Scr). Western blotting was performed using an anti-CCDC88A antibody. **b**, **c**. Oligonucleotides targeting *CCDC88A* or Scr were transiently transfected into S2-013 and PANC-1 cells. Migration (**b**) and two-chamber invasion assays (**c**) were performed. Migrating cells in four fields per group were scored. Data are derived from three independent experiments. *Columns*, mean; *bars*, SD. *p < 0.005 compared with Scr-transfected control (Student’s *t*-test). **d**, **e**. Western blots (**d**) and confocal immunofluorescence microscopic images (**e**). Mock control vector or a myc-tagged CCDC88A-rescue construct was transiently transfected into scrambled control-siRNA and *CCDC88A*-siRNA transfected S2-013 and PANC-1 cells; after 48 h, the cells were incubated on fibronectin for 5 h. Western blots probed with anti-myc and anti-CCDC88A antibodies are shown. Cells were stained with anti-myc antibody (*green*). Actin filaments were labeled with phalloidin (*red*). Arrows, myc-tagged CCDC88A localized in cell protrusions. Blue, DAPI staining. Bar, 10 μm. **f**, **g**. Mock control vector or a myc-tagged CCDC88A-rescue construct was transiently transfected into scrambled control-siRNA and *CCDC88A*-siRNA transfected S2-013 and PANC-1 cells; 48 h later, migration (**f**) and two-chamber invasion assays (**g**) were performed. Migrating cells in four fields per group were counted. Data are derived from three independent experiments. *Columns*, mean; *bars*, SD. *p < 0.005 compared with corresponding *CCDC88A*-siRNA transfected S2-013 cells that were transfected with mock vector (Student’s *t*-test)

To determine whether CCDC88A participated in the migration and invasiveness of PDAC cells, CCDC88A expression was transiently suppressed by transfection of *CCDC88A*-specific small interfering RNA (siRNA) oligos into S2-013 and PANC-1 cells. Western blot data indicated that, at 48 h after transfection, the expression of CCDC88A was markedly higher in scrambled control-siRNA transfected S2-013 and PANC-1 cells than in the corresponding *CCDC88A*-siRNA transfected cells (Fig. 3a). Suppression of CCDC88A did not affect cell growth of S2-013 or PANC-1 cells in an in vitro MTT assay (data not shown), but it did inhibit cell migration of both cells in migration assays (Fig. 3b). Furthermore, in two-chamber invasion assays, *CCDC88A*-siRNA transfected cells were significantly less invasive than the control-siRNA transfected S2-013 and PANC-1 cells (Fig. 3c). When a CCDC88A-rescue construct was transfected into *CCDC88A*-siRNA transfected S2-013 cells, the exogenous CCDC88A expressed from the rescue construct was localized in the cytoplasm of cell bodies and in cell protrusions, similar to endogenous CCDC88A (Fig. 3d, e). The transfection of a CCDC88A-rescue construct into *CCDC88A*-siRNA transfected S2-013 and PANC-1 cells abrogated the changes to cell migration and invasiveness caused by the *CCDC88A*-siRNA (Fig. 3f, g). These results indicated that endogenous CCDC88A specifically promotes PDAC cell migration and invasion.

### Co-localization of CCDC88A and actin-filaments in cell protrusions

**Fig. 4:**
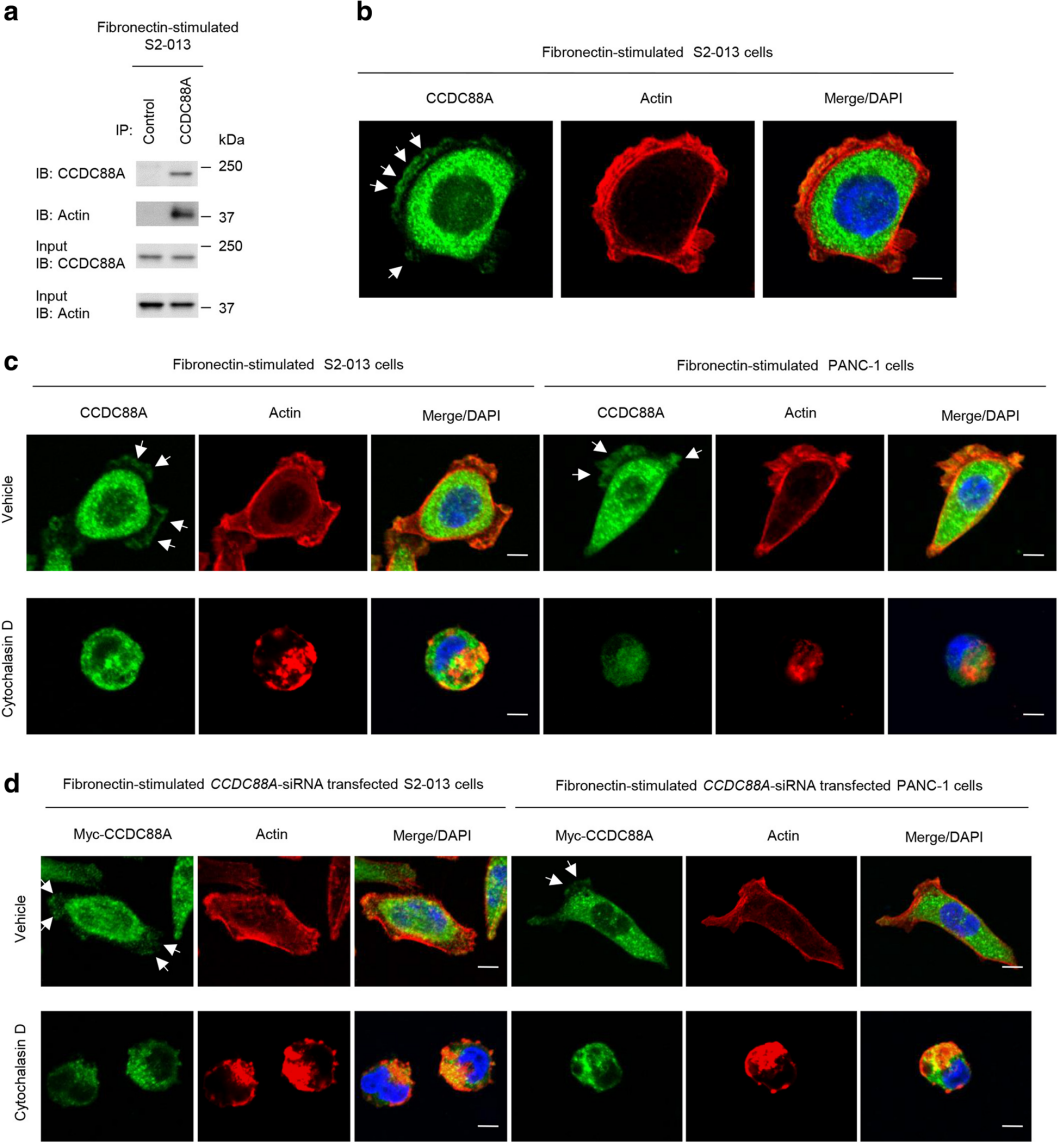
Co-localization of CCDC88A with actin-filaments in cell protrusions. **a**. Immunoprecipitation (IP) of CCDC88A from S2-013 cells cultured on fibronectin. Proteins within the immunoprecipitates were examined by western blotting. The blots were probed with antibodies against CCDC88A and actin. Mouse IgG isotype control antibody was used as an isotype control. **b**. Confocal immunofluorescence microscopic images show nuclear DAPI staining (*blue*), abundant cytoplasmic CCDC88A, and the accumulation of CCDC88A (*green*) in membrane protrusions of fibronectin-stimulated S2-013 cells. Actin filaments were labeled with phalloidin (*red*). Arrows, CCDC88A that was colocalized with actin-filaments in cell protrusions. Bar, 10 μm. **c**. Confocal immunofluorescence microscopic images of S2-013 and PANC-1 cells that were pretreated with 100 μM Cytochalasin D for 12 h and were then incubated on fibronectin. Cells were stained with anti-CCDC88A antibody (*green*). Actin filaments were labeled with phalloidin (*red*). Arrows, CCDC88A that was colocalized with actin-filaments in cell protrusions. Blue, DAPI staining. Bars, 10 μm. **d**. Confocal immunofluorescence microscopic images. *CCDC88A*-siRNA transfected S2-013 and PANC-1 cells, which were transiently transfected with a myc-tagged CCDC88A-rescue construct, were pretreated with 100 μM Cytochalasin D for 12 h, and were subsequently incubated on fibronectin. Cells were stained with anti-myc antibody (*green*). Actin filaments were labeled with phalloidin (*red*). Arrows, CCDC88A that was colocalized with actin-filaments in cell protrusions. Blue, DAPI staining. Bars, 10 μm

To determine if CCDC88A co-localized with actin, CCDC88A was immunoprecipitated (IP) from lysates of fibronectin-stimulated S2-013 cells and an anti-actin antibody was used to detect filamentous actin in multiprotein complexes that were precipitated by the anti-CCDC88A antibody. A strong actin band was detected in immunoblots of the anti-CCDC88A-immunoprecipitates (Fig. 4a), and actin was enriched in CCDC88A-IPs compared to control IgG-IPs. Immunofluorescence analysis showed that CCDC88A was associated with peripheral actin structures in cell protrusions of fibronectin-stimulated S2-013 cells (Fig. 4b). These results suggested that CCDC88A is an actin-binding protein that is present in cell protrusions of PDAC cells.

To determine whether alteration of actin cytoskeleton dynamics could directly affect the subcellular distribution of CCDC88A, we treated S2-013 and PANC-1 cells with the actin depolymerising agent Cytochalasin D. There were fewer peripheral actin structures in S2-013 and PANC-1 cells exposed to 100 μM Cytochalasin D for 12 h than in non-treated cells and CCDC88A was localized in the cytoplasm in the treated cells (Fig. 4c). Transfection of a CCDC88A-rescue construct into *CCDC88A*-siRNA transfected S2-013 and PANC-1 cells increased peripheral actin structures and co-localization of the exogenous CCDC88A with peripheral actin structures in cell protrusions of non-treated cells was observed; however, such exogenous CCDC88A did not increase the formation of peripheral actin structures in Cytochalasin D-treated *CCDC88A*-siRNA transfected S2-013 and PANC-1 cells, and exogenous CCDC88A displayed an intracellular localization in those cells (Fig. 4d).

### Roles of CCDC88A in the formation of cell protrusions

**Fig. 5:**
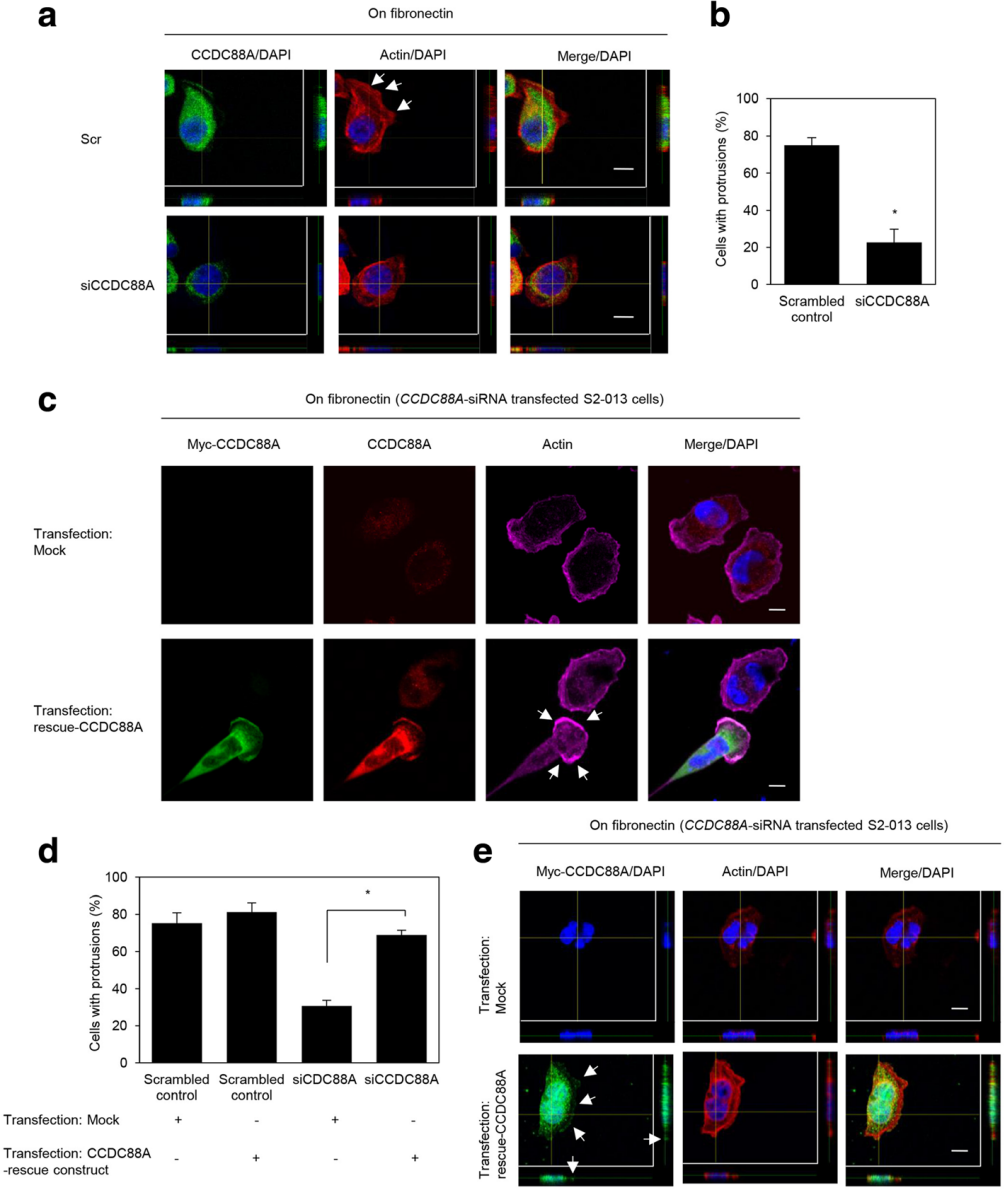
Roles of CCDC88A in the formation of cell protrusions. **a**. Confocal Z stack images of S2-013 cells that were transiently transfected with scrambled control-siRNA (Scr) or *CCDC88A*-siRNA (siCCDC88A). The transfected cells were incubated on fibronectin, and were subsequently stained with anti-CCDC88A antibody (*green*) and phalloidin (*red*). The lower and right panels in the confocal Z stack show a vertical cross-section (*yellow lines*) through the cells. Arrows, peripheral actin structures in cell protrusions of control-siRNA transfected cells. Blue, nuclear DAPI staining. Bars, 10 μm. **b**. Quantification of the data shown in Fig. 5a. *Columns*, mean; *bars*, SD. *p < 0.001 compared with Scr-transfected controls (Student’s *t*-test). **c**. Confocal immunofluorescence microscopic images of S2-013 cells that had been transfected with *CCDC88A*-siRNA and were subsequently transfected with a myc-tagged CCDC88A-rescue construct. After 48 h, the cells were incubated on fibronectin. Cells were stained with anti-myc antibody (green), anti-CCDC88A antibody (red) and phalloidin (violet). Arrows, cell protrusions reproduced by myc-tagged CCDC88A in *CCDC88A*-siRNA transfected cells. Blue, DAPI staining. Bars, 10 μm. **d**. Quantification of the data shown in Fig. 5c; the values represent the number of cells with fibronectin-mediated cell protrusions in which peripheral actin structures were increased. All cells in four fields per group were scored. Data are derived from three independent experiments. *Columns*, mean; *bars*, SD. *p < 0.001 compared with corresponding *CCDC88A*-siRNA transfected S2-013 cells that were transfected with mock vector (Student’s *t*-test) **e**. Confocal Z stack images showing nuclear DAPI staining (*blue*) and the accumulation of myc-tagged CCDC88A (*green*) in fibronectin-stimulated CCDC88A-siRNA transfected S2-013 cells transfected with the myc-tagged CCDC88A-rescue construct. Arrows, myc-tagged CCDC88A accumulated in cell protrusions. The lower and right panels of the confocal Z stack show a vertical cross-section (*yellow lines*) through the cells. Bar, 10 μm

To determine whether CCDC88A participated in the induction of membrane protrusions, we analyzed peripheral actin structures in membrane ruffles of fibronectin-stimulated control scrambled siRNA-transfected S2-013 and PANC-1 and *CCDC88A-*siRNA transfected S2-013 and PANC-1 cells. Confocal microscopy showed that *CCDC88A*-knockdown decreased peripheral actin structures, compared to control S2-013 and PANC-1 cells (Fig. 5a, S2-013 cells; Additional file 1: Figure S1a, PANC-1 cells). Furthermore, *CCDC88A*-knockdown significantly inhibited fibronectin-mediated formation of membrane protrusions, compared to control S2-013 and PANC-1 cells (Fig. 5b, S2-013 cells; Additional file 1: Figure S1b, PANC-1 cells). Transfection of a CCDC88A-rescue construct into *CCDC88A*-siRNA transfected S2-013 and PANC-1 cells abrogated the decrease in membrane ruffles, which are peripheral actin structures (Fig. 5c and d, S2-013 cells; Additional file 1: Figure S1c and d, PANC-1 cells) that was caused by *CCDC88A*-siRNA. Since the exogenous CCDC88A from the rescue construct was strongly accumulated in cell protrusions and co-localized with actin-filaments in the protrusions of *CCDC88A*-siRNA transfected S2-013 and PANC-1 cells (Fig. 5c and e, S2-013 cells; Additional file 1: Figure S1c and e, PANC-1 cells), CCDC88A that is localized in cell protrusions could play a role in the formation of membrane protrusions in PDAC cells.

### Roles of CCDC88A in regulation of the activation of Akt

**Fig. 6:**
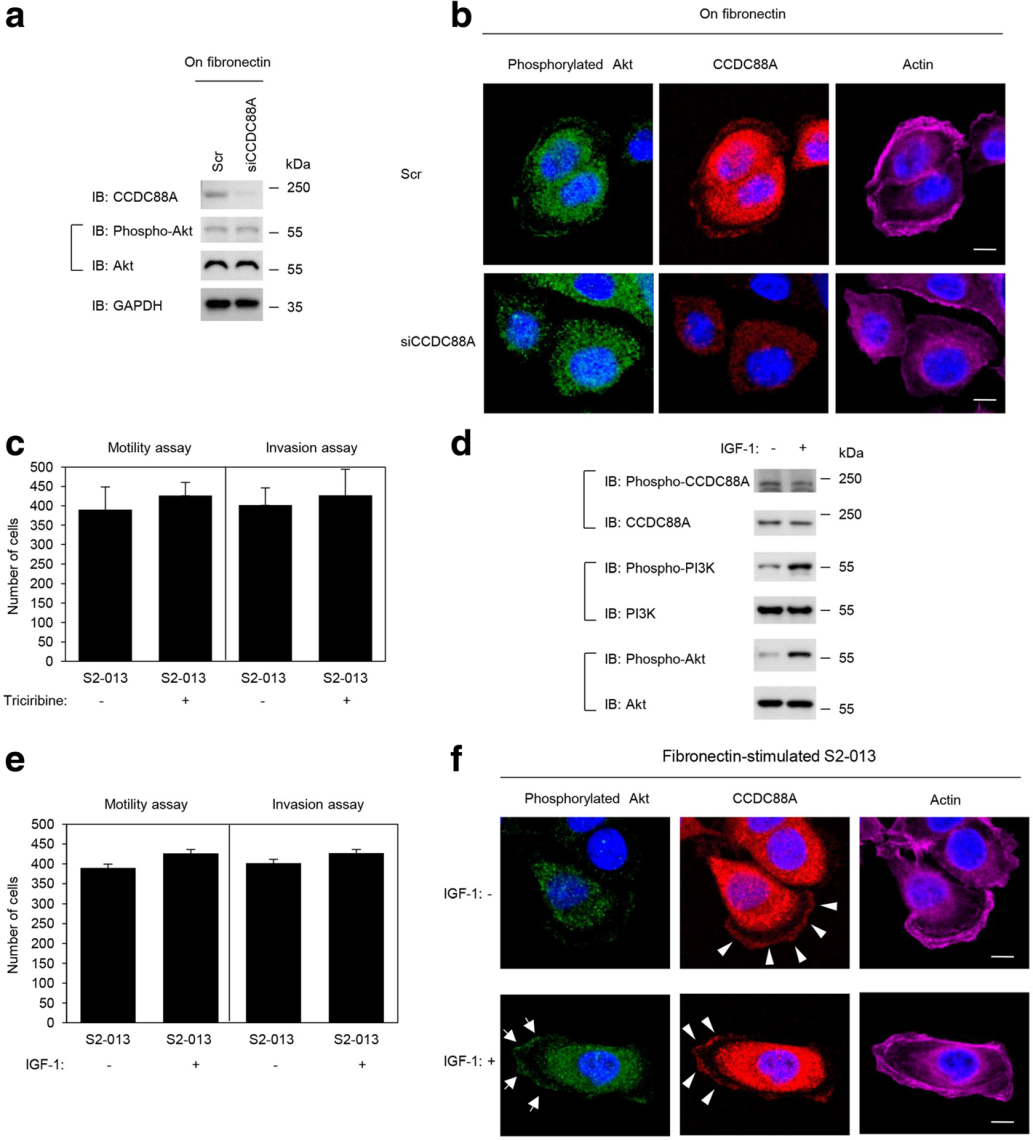
Roles of CCDC88A in regulating the activation of Akt. **a**. Scr or siCCDC88A oligos were transfected into S2-013 cells. After 48 h, the cells were incubated on fibronectin for 5 h, and whole cell lysates were prepared for western blot analysis using anti-CCDC88A, anti-Akt and anti-phosphorylated Akt antibodies. Data are representative of three independent experiments. **b**. Confocal immunofluorescence microscopic images of S2-013 cells that were transiently transfected with scrambled control-siRNA or *CCDC88A*-siRNA. After 48 h, the cells were incubated on fibronectin for 5 h and were then stained with anti-phosphorylated Akt antibody (*green*), anti-CCDC88A antibody (*red*) and phalloidin (*violet*; *actin filaments*). Blue, nuclear DAPI staining. Bars, 10 μm. **c**. S2-013 cells were pretreated with or without 5 μM of the Akt inhibitor triciribine for 2 h, following which the cells were plated on migration (*left panel*) and Matrigel invasion (*right panel*) chambers. Migrated cells in four fields per group were counted. Data are representative of three independent experiments. *Columns*, mean; *bars*, SD. **d**. S2-013 cells were pretreated with or without 100 ng/mL IGF-1 for 10 min, and whole cell lysates were prepared for western blot analysis using anti-CCDC88A, anti-phosphorylated CCDC88A, anti-PI3K, anti-phosphorylated PI3K, anti-Akt, and anti-phosphorylated Akt antibodies. Data are representative of three independent experiments. **e**. S2-013 cells were pretreated with or without 100 ng/mL IGF-1 for 10 min and the cells were then plated on migration (*left panel*) and Matrigel invasion (*right panel*) chambers. Migrated cells in four fields per group were counted. Data are representative of three independent experiments. *Columns*, mean; *bars*, SD. **f**. Confocal immunofluorescence microscopic images of S2-013 cells that were cultured on fibronectin with or without IGF-1 stimulation following which the cells were stained with anti-phosphorylated Akt antibody (*green*), anti-CCDC88A antibody (*red*) and phalloidin (*violet*; *actin*). Arrows, phosphorylated Akt accumulated in cell protrusions; arrowheads, CCDC88A accumulated in cell protrusions. Blue, nuclear DAPI staining. Bars, 10 μm

To determine whether CCDC88A was associated with the regulation of Akt phosphorylation in PDAC cells, we performed western blot and immunocytochemical analyses of fibronectin-stimulated scrambled control-siRNA transfected S2-013 and PANC-1 and *CCDC88A*-siRNA transfected S2-013 and PANC-1 cells. Phosphorylated Akt (Ser-473) in the total cell lysates was unchanged between cells with scrambled control and cells with knockdown of CCDC88A (Fig. 6a, S2-013 cells; Additional file 2: Figure S2a, PANC-1 cells). Phosphorylated Akt is known to be recruited to the plasma membrane by an increase in PI3K signaling [18]. Immunocytochemical analysis showed that the subcellular distribution of phosphorylated Akt (Ser-473) was not different between fibronectin-stimulated control-siRNA transfected S2-013 or PANC-1 and *CCDC88A-*siRNA transfected S2-013 or PANC-1 cells (Fig. 6b, S2-013 cells; Additional file 2: Figure S2b, PANC-1 cells). It should be noted that exogenous CCDC88A was still expressed in the cytoplasm of cell bodies, but not at cell protrusions in *CCDC88A-*siRNA transfected S2-013 and PANC-1 cells. Addition of the Akt inhibitor triciribine (5 μM for 2 h) to the culture medium of S2-013 and PANC-1 cells did not change the migration or invasiveness of S2-013 and PANC-1 cells compared with non-treated cells (Fig. 6c, S2-013 cells; Additional file 2: Figure S2c, PANC-1 cells). Since MTT analysis showed that triciribine did not induce growth inhibition of S2-013 and PANC-1 cells at concentrations of up to 5 μM (data not shown), a concentration of 5 μM was used to study the effect of triciribine on cell migration and invasiveness. Furthermore, it has been shown that the binding of IGF-1 to its receptor triggers the activation of PI3K [19, 20]. We therefore cultured S2-013 and PANC-1 cells in medium alone (control) or in medium containing IGF-1 (100 ng/ml for 10 min), and the migration and invasion of these cells were assayed. Although IGF-1 treatment activated PI3K and Akt, it did not increase the level of phosphorylated CCDC88A (Fig. 6d, S2-013 cells; Additional file 2: Figure S2d, PANC-1 cells). Moreover, IGF-1 treatment did not change the migration or invasiveness of S2-013 and PANC-1 cells, compared with non-treated cells (Fig. 6e, S2-013 cells; Additional file 2: Figure S2e, PANC-1 cells). IGF-1 stimulation did induce the translocation of phosphorylated Akt towards the plasma membranes of S2-013 and PANC-1 cells; however, it did not change the intracellular distribution of CCDC88A (Fig. 6f, S2-013 cells; Additional file 2: Figure S2f, PANC-1 cells). These results suggested that endogenous CCDC88A was not associated with modulation of the activity of Akt, and that Akt signaling was not necessary for the migration or invasion of PDAC cells.

### Association of CCDC88A and AMPK1 with the formation of cell protrusions

**Fig. 7:**
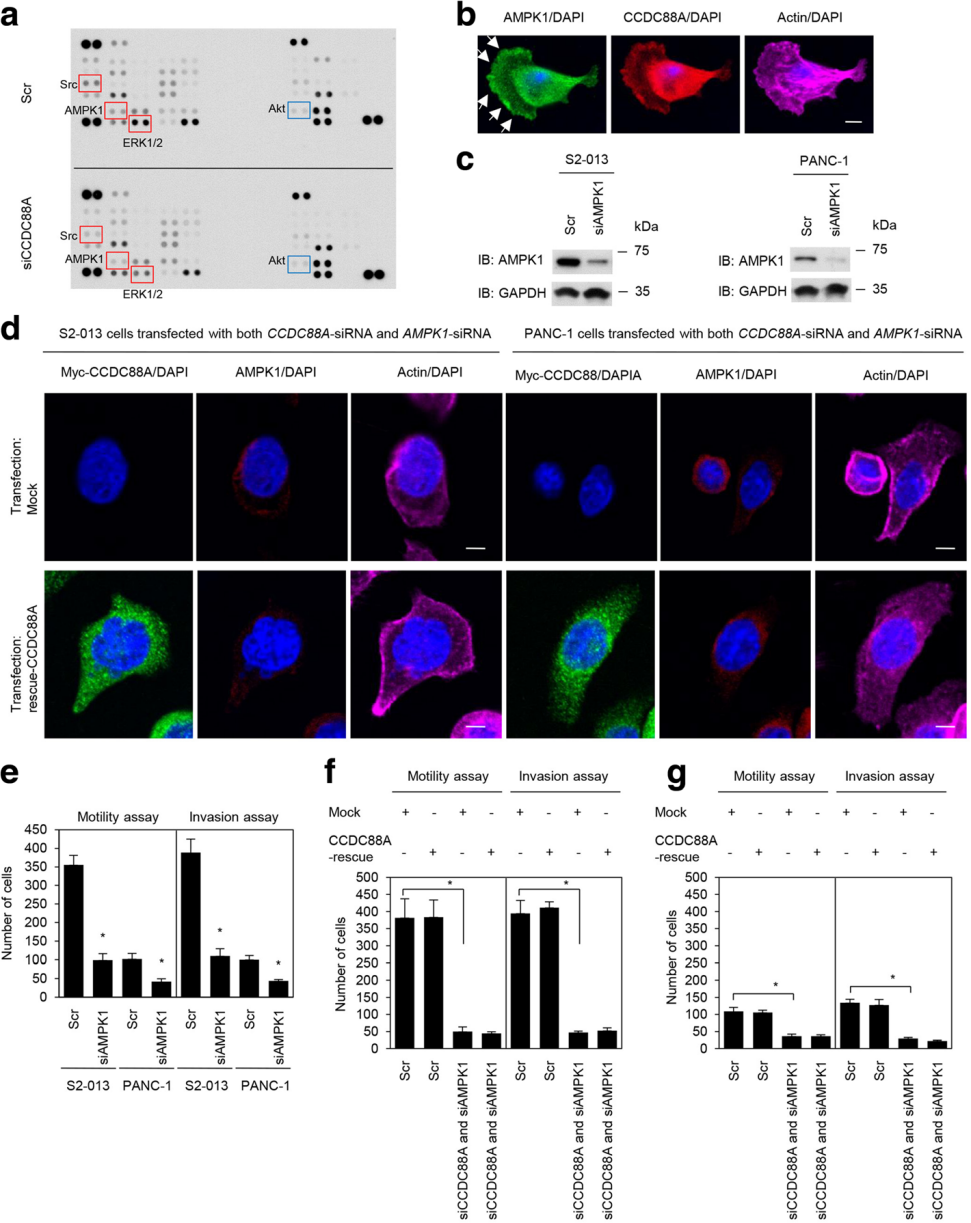
Association of CCDC88A and AMPK1 with cell migration and invasion. a. Effects of suppression of CCDC88A on the expression of selected phosphoproteins in S2-013 cells. Cell extracts obtained from fibronectin-stimulated scrambled control-siRNA transfected S2-013 cells or *CCDC88A*-siRNA transfected S2-013 cells were probed on human phosphoprotein arrays. **b**. Confocal immunofluorescence microscopic images of S2-013 cells that were cultured on fibronectin and were then labeled with anti-AMPK1 antibody (*green*), anti-CCDC88A antibody (*red*) and phalloidin (violet; actin filaments). Arrows, AMPK1 localized in cell protrusions. Blue, nuclear DAPI staining. Bar, 10 μm. **c**. Western blot analysis of AMPK1 following transient transfection of S2-013 and PANC-1 cells with a single mixture containing four different siRNA oligonucleotides targeting *AMPK1* (siAMPK1) or negative scrambled control (Scr). Western blotting was performed using an anti-AMPK1 antibody. **d**. Confocal immunofluorescence microscopic images. A myc-tagged CCDC88A-rescue construct was transfected into S2-013 and PANC-1 cells that had been transfected with both *CCDC88A*-siRNA and *AMPK1*-siRNA. 48 h later, the cells were incubated on fibronectin. Cells were stained with anti-myc antibody (*violet*), anti-AMPK1 antibody (*green*), and phalloidin (*red*). Blue, DAPI staining. Bars, 10 μm. **e**. siRNA oligonucleotides targeting *AMPK1* or Scr were transiently transfected into S2-013 and PANC-1 cells. After 48 h, migration and two-chamber invasion assays were performed. Migrating cells in four fields per group were scored (*lower panel*). Data are representative of three independent experiments. *Columns*, mean; *bars*, SD. *p < 0.008 compared with Scr-transfected control (Student’s *t*-test). **f**, **g**. A myc-tagged CCDC88A-rescue construct was transfected into S2-013 (**f**) and PANC-1 (**g**) cells that had been transfected with *CCDC88A*-siRNA and *AMPK1*-siRNA; 48 h later, migration and two-chamber invasion assays were performed. Migrating cells in four fields per group were counted. Data are derived from three independent experiments. *Columns*, mean; *bars*, SD. *p < 0.005 compared with corresponding *CCDC88A*-siRNA and *AMPK1*-siRNA transfected cells that were transfected with mock vector (Student’s *t*-test)

We further analyzed intracellular signaling pathways other than the IGF-1-PI3K-Akt pathway in the scrambled control-siRNA transfected and *CCDC88A*-siRNA transfected S2-013 cells (Fig. 7a). We used a commercially available human phosphoprotein array kit that allowed semi-quantitative assessment of the levels of phosphorylated representatives of a number of signaling pathways including MAP kinase, the Src family, and JAK/signal transducer and activator of transcription (STAT) pathways. Consistent with the earlier findings, phosphorylated Akt was unchanged between scrambled control-siRNA transfected S2-013 cells and *CCDC88A*-siRNA transfected S2-013 cells. Of the 38 kinases investigated, suppression of CCDC88A resulted in the inactivation of Src and extracellular signal-regulated kinase (ERK) 1/2, and the activation of adenosine monophosphate-activated protein kinase 1 (AMPK1). Immunofluorescent analysis of fibronectin-stimulated S2-013 and PANC-1 cells indicated that, in addition to CCDC88A, AMPK1 also accumulated in cell protrusions (Fig. 7b, S2-013 cells; Additional file 3: Figure S3, PANC-1 cells).

The high expression of AMPK1 in S2-013 and PANC-1 cells was transiently suppressed by *AMPK1*-specific siRNA oligos (Fig. 7c). Western blot analysis indicated that, 48 h after transfection, the expression of AMPK1 was much higher in scrambled control siRNA transfected S2-013 and PANC-1 cells than in the corresponding *AMPK1*-siRNA transfected cells. Interestingly, transfection of a CCDC88A-rescue construct into S2-013 and PANC-1 cells in which both endogenous CCDC88A and AMPK1 had been suppressed did not reproduce cell protrusions in which peripheral actin-filaments were assembled (Fig. 7d). Moreover, transfection of a CCDC88A-rescue construct into S2-013 and PANC-1 cells in which both endogenous CCDC88A and AMPK1 had been suppressed did not induce the accumulation of exogenous CCDC88A at peripheral actin structures (Fig. 7d).

### Association of CCDC88A and AMPK1 with the cell migration and invasion of PDAC cells

Trans-well migration and Matrigel invasion assays, and siRNA-mediated knockdown were used to examine the effect of AMPK1 on the migration and invasiveness of S2-013 and PANC-1 cells. In the trans-well migration assays, the migration of both S2-013 and PANC-1 cells was significantly lower in *AMPK1*-knockdown cells than in control cells (Fig. 7e). In two-chamber invasion assays, the invasiveness of S2-013 and PANC-1 cells was significantly lower in *AMPK1*-knockdown cells than in control cells (Fig. 7e). Moreover, transfection of a CCDC88A-rescue construct into S2-013 and PANC-1 cells in which both endogenous CCDC88A and AMPK1 expression had been suppressed did not abrogate the changes to cell migration and invasiveness caused by the *CCDC88A*-siRNA and *AMPK1*-siRNA (Fig. 7f, S2-013 cells; Fig. 7g, PANC-1 cells). These results indicated that AMPK1 was necessary for the CCDC88A-associated promotion of migration and invasiveness of PDAC cells.

## Discussion

In this study, we showed that CCDC88A is a significant prognostic factor whose expression levels predict the overall survival of patients with PDAC. Individual clinicopathological factors were not significantly correlated with CCDC88A expression, which may be due to the remarkably short overall survival of patients with PDACs (Table 2). The important fact that patients with high CCDC88A expression showed significantly worse overall survival in both univariate and multivariate analyses suggested that high CCDC88A expression might be a novel and independent prognostic factor of PDAC (Table 3). Immunocytochemical analysis of the present study indicated that CCDC88A accumulated in cell protrusions of PDAC cells. Immunohistochemical analysis additionally showed that CCDC88A was more abundantly localized in the basolateral portions of PDAC cells in the high-expressing CCDC88A group, compared to the low-expressing CCDC88A group (Fig. 1b). PDAC is one of the deadliest of cancers due to its ability to extensively invade surrounding tissues and to metastasize at an early stage [10]. Extensive local infiltration and metastasis are the main causes of death in PDAC [21]. Since the formation of cell protrusions is essential for cell migration and invasion, we investigated the role of CCDC88A in the promotion of migration and invasiveness of PDAC cells through the formation of cell protrusions. Moreover, we confirmed that CCDC88A was clearly not expressed in normal organs such as pancreas, brain, lung, liver, and kidney. Consequently, CCDC88A is an essential marker of poor prognosis that is functionally related to cell migration and invasion through promotion of an increase in the formation of cell protrusions.

CCd for enhancement of the activity of Akt, the remodeling of actin, and the triggering of cell migration by growth factors such as epidermal growth factor (EGF), IGF, vascular endothelial growth factor (VEGF) and insulin [1, 5, 9]. The reported mechanism by which the EGF receptor enhances the activity of Akt via CCDC88A is that the carboxy terminus of CCDC88A directly binds the autophosphorylated cytoplasmic tail of the EGF receptor and thereby links G-protein to ligand-activated receptors [21]. The carboxy terminus of CCDC88A also serves as a common platform that links ligand-activated receptors at the leading edge to actin, Akt, and Gαi, 3 whose interplay is essential for cell migration [1, 5, 9, 22]. Furthermore, in response to migratory cues such as growth factors, phosphorylation of CCDC88A by Akt occurs at the leading edge and this phosphorylation is required for directional cell migration that, in the case of cancer cells, ultimately leads to cell invasion and metastasis [7]. Thus, CCDC88A functions as a signal transducer in the PI3-K/Akt signaling pathway, and plays important roles in Akt-dependent cell invasion and metastasis. We showed that IGF-1 stimulation that induced the translocation of phosphorylated Akt towards the plasma membrane did not increase the level of phosphorylated CCDC88A (Fig. 6d) or alter the intracellular distribution of CCDC88A in PDAC cells (Fig. 6f). We suggest that CCDC88A assembled in cell protrusions in PDAC cells, where it co-localized with actin-filaments, but that the level of active Akt was not associated with the translocation of CCDC88A towards cell protrusions (Fig. 6b). Additionally, treatment of fibronectin-stimulated PDAC cells with the Akt inhibitor triciribine did not change the formation of cell protrusions (data not shown) or cell migration or invasiveness (Fig. 6c, Additional file 2: Figure S2c). Our in vitro assays showed that neither the phosphorylation level of CCDC88A nor CCDC88A-dependent cell invasion were associated with the phosphorylation level of Akt in PDAC cells. Our results therefore suggested that CCDC88A functioned in the promotion of cell migration and invasion without association with Akt-signaling pathways in PDAC cells.

We showed that knockdown of CCDC88A decreased peripheral rearrangements of the actin cytoskeleton in PDAC cells cultured on fibronectin, and that exogenously overexpressed CCDC88A significantly increased peripheral actin-cytoskeletal rearrangements in *CCDC88A*-siRNA transfected PDAC cells. CCDC88A localizes to the actin cytoskeleton including to stress fibers (actin microfilament bundles) and cortical actin filaments where it can bind to both the plasma membrane and actin filaments [1]. Consistent with the results of a previous report [1], treatment with Cytochalasin D decreased the co-localization of CCDC88A with actin filaments in cell protrusions of PDAC cells (Fig. 4c). Moreover, transfection of a CCDC88A-rescue construct into PDAC cells in which endogenous CCDC88A expression was suppressed failed to increase peripheral actin-cytoskeletal rearrangements in Cytochalasin D treated cells (Fig. 4d). The fact that CCDC88A remained in the cytoplasm of cell bodies of *CCDC88A*-siRNA transfected PDAC cells (Fig. 5a and 6b), suggested that it is the CCDC88A that accumulates at the cell membranes that contributes to peripheral actin-cytoskeletal rearrangements. Dynamic actin remodeling processes at the leading edge of migrating cells are complex and involve increased actin filament severing, capping and dendritic branching [23]. Migratory competence of tumor cells requires activation of the motile cycle, the first step of which is actin remodeling, which drives the formation of cell protrusions, defines the direction of migration and initiates the growth of the lamellipodia [24]. Given that CCDC88A played a role in regulating peripheral actin-rearrangements, the observation that CCDC88A co-localized with actin-filaments in cell protrusions indicated that CCDC88A must contribute to the regulation of membrane ruffles, resulting in the promotion of PDAC cell migration and invasion. The mechanism by which CCDC88A promotes cell migration and invasion thorough the regulation of membrane protrusions in PDAC cells is still unknown. We found that CCDC88A knockdown inactivated Src and ERK1/2 in S2-013 cells (Fig. 7a). Src is at the cross-roads of several signaling pathways, including Ras/Raf/ERK1/2, PI3K/Akt, and STAT 3 pathways [24–26], resulting in the regulation of cell proliferation, survival, invasion, migration and angiogenesis. Furthermore, CCDC88A knockdown activated the highly conserved, energy-sensing serine/threonine kinase AMPK1 (Fig. 7a). Interestingly, AMPK1 also accumulated in cell protrusions of fibronectin-stimulated PDAC cells (Fig. 7b). Knockdown of AMPK1 inhibited PDAC cell migration and invasion (Fig. 7e). Transfection of a CCDC88A-rescue construct into PDAC cells in which both CCDC88A and AMPK1 were suppressed failed to increase the formation of cell protrusions, or cell migration and invasion (Fig. 7d, f and g). These findings suggested that AMPK1 pathways might be associated with CCDC88A-dependent migration and invasiveness. Although many studies support a tumor-suppressive role of AMPK [27, 28], emerging evidence suggests that this function of AMPK1 might be overridden by stress or oncogenic signals so that, under such conditions, tumor cells use AMPK activation as a survival strategy to gain growth advantage [29]. Future studies should evaluate the detailed CCDC88A-associated signaling cascades that coordinate the actin-cytoskeletal remodeling that is required for cell spreading and cell migration and invasion.

## Conclusions

The findings presented in this study are supportive of the pivotal roles of CCDC88A in the coordinated regulation of cortical actin changes. The functional significance of CCDC88A, which mediates migration and invasiveness without association with Akt signaling in PDAC cells, was established. The data presented here indicated that inhibition of CCDC88A may be effective for targeted molecular therapy, because any such therapy would limit the migration and invasiveness of PDACs.

## Methods

### Primary human PDAC samples

Patients (*n* = 102) who underwent surgical treatment for PDAC at the Departments of Surgery, Kochi Medical School Hospital (Nankoku, Japan) and Matsuyama Shimin Hospital (Matsuyama, Japan) between 1999 and 2014 were studied (clinicopathological findings of these 102 patients are summarized in Table 1. The follow-up period for survivors ranged from 18 to 192 months (mo) (median, 64.0 mo). Of these patients, 83 had received adjuvant chemotherapy with gemcitabine or S-1, or chemoradiation therapy after resection of the PDAC. Tumors were classified according to the classification of pancreatic carcinoma of the Japan Pancreas Society [30] and the UICC TNM classification [31].

### Antibodies

Anti-CCDC88A antibody (MABT100) and the JLA20 anti-actin antibody (MABT219) were purchased from Merck Millipore (Temecula, CA). Anti-phosphorylated CCDC88A (Ser1416) antibody (28067) was purchased from Immuno-Biological Laboratories (Gunma, Japan). Anti-phosphorylated PI3K (Tyr458/Tyr199) (4228), anti-PI3K (pan) (4257), anti-phosphorylated Akt (Ser473) (9271), and anti-Akt (pan) (4691) antibodies were purchased from Cell Signaling (Grand Island, NY). Anti-AMPK1 antibody (A300-507A) was purchased from Bethyl Laboratories (Montgomery, TX). Anti-myc (A14) antibody was purchased from Santa Cruz Biotechnology (Santa Cruz, CA).

### Immunohistochemical staining

Tissue sections from normal pancreas, brain, lung, liver and kidney were purchased from Biochain (Hayward, CA). The sections were deparaffinized and autoclaved at 108 °C for 15 min. After endogenous peroxidase activity was quenched by incubation for 30 min in 0.33% hydrogen peroxide diluted in methanol, the sections were blocked by incubation with fetal bovine serum. Sections were then incubated with anti-CCDC88A antibody at room temperature for 1 h and were subsequently washed with PBS. Immunodetection was performed with peroxidase-labeled anti-rabbit immunoglobulin (Dako Cytomation, Carpinteria, CA). Finally, the reactants were developed with 3,3′-diaminobenzidine (Dako), and the sections were counterstained with hematoxylin.

### Evaluation of CCDC88A staining

CCDC88A staining was evaluated by two independent observers (SN and MF) who were blinded to clinical and outcome data. Immunoreactivity was scored semiquantitatively according to the estimated percentage of positive tumor cells (1, <50%; 2, 50-80%; 3, >80% reactive cells) and intensity (1, weaker; 2, equal to; and 3, stronger than the staining intensity of the islet of Langerhans). The slides whose islet of Langerhans was not significantly stained, were considered to be in bad condition and were not evaluated. A total immunohistochemical score was calculated by summing the percentage score and the intensity score. The quantity of CCDC88A expression was classified into two groups based on the total score (low group, 2–3; high group, 4–6) with reference to a previous report [13].

### Cell culture

The human PDAC cell line S2-013, a subline of SUIT-2, was obtained from Dr. T. Iwamura (Miyazaki Medical College, Miyazaki, Japan) [32]. The human PDAC cell line PANC-1 was purchased from the American Type Culture Collection (Manassas, VA). All cells were grown in Dulbecco’s modified Eagle’s medium (DMEM; Gibco-BRL, Carlsbad, CA) supplemented with 10% heat-inactivated fetal calf serum (FCS) at 37 °C in a humidified atmosphere saturated with 5% CO_2_. In selected experiments, cell suspensions were cultured with IGF-1 (100 ng/mL; Sigma-Aldrich, St. Louis, MO) for 10 min and the Akt inhibitor triciribine (5 μM; Sigma-Aldrich) for 2 h.

### Confocal immunofluorescence microscopy

Coverslips were coated with 10 μg/mL fibronectin (Sigma-Aldrich) for 1 h at room temperature. Cells were seeded on fibronectin-coated glass coverslips and incubated for 5 h. The cells were then fixed with 4% paraformaldehyde, permeabilized with 0.1% Triton X-100, covered with blocking solution (3% BSA/PBS), and then incubated with the primary antibody for 1 h. Alexa488- or Alexa594-conjugated secondary antibody (Molecular Probes, Carlsbad, CA) was then applied with or without Alexa647-conjugated phalloidin (Cytoskeleton, Denver, CO). In some experiments, a commercial antibody-labeling technology (Zenon; Life Technologies, Carlsbad, CA) was used according to the manufacturer’s instructions to conjugate green or red fluorophores to primary antibodies. Each specimen was visualized using a Zeiss LSM 510 META microscope (Carl Zeiss, Gottingen, Germany).

### siRNA treatment

A single mixture with four different siRNA oligonucleotides (oligos) targeting *CCDC88A* or *AMPK1* was purchased from Qiagen (FlexiTube GeneSolution siRNA GS55704 and GS5562, respectively; Valencia, CA) and a single mixture with four different scrambled negative control siRNA oligos was obtained from Santa Cruz (37007). To examine the effect of the siRNAs on CCDC88A expression, S2-013 and PANC-1 cells that expressed CCDC88A were plated in six-well plates. After 20 h, the cells were transfected with 80 pmol of each siRNA mixture in siRNA transfection reagent (Qiagen) following the manufacturer’s instructions. After incubation for 48 h, the cells were processed for western blotting or for transwell migration or Matrigel invasion assays.

### CCDC88A-rescue construct

Reverse transcription-PCR (RT-PCR) was used to amplify the entire coding sequence of the *CCDC88A* cDNA. The resultant PCR product was subsequently inserted into a separate pCMV6-Entry vector (Origene Technologies, Rockville, MD) bearing a C-terminal myc-DDK-tag. The X-tremeGENE HP DNA Transfection Reagent (Roche, Penzberg, Germany) was used to transiently transfect target cells with the resultant *CCDC88A*-rescue construct.

### Immunoprecipitation

S2-013 cells were seeded onto fibronectin and incubated for 5 h. The cells were then lysed in lysis buffer (20 mM HEPES (pH 7.4), 100 mM KCl, 5 mM MgCl_2_, 0.5% Triton X-100, protease inhibitor cocktail tablets (Roche) and a phosphatase inhibitor cocktail (Nacalai, Kyoto, Japan)). Lysates were immunoprecipitated with Dynabeads Protein G (Dynal, Oslo, Norway) and with anti-CCDC88A antibody or mouse IgG isotype control antibody for 2 h at 4 °C. Beads were pelleted using a magnetic rack (Dynal) and were subsequently analyzed using western blotting.

### Immunoblot analysis of cell lysates

Each cell pellet was resuspended in a buffer containing 20 mM Hepes (pH 7.4), 100 mM KCl, 2 Mm MgCl_2_, 0.5% Triton X-100, protease inhibitor cocktail tablets (Roche) and a phosphatase inhibitor cocktail (Nacalai, Kyoto, Japan). The bicinchoninic acid (BCA) assay was used to determine protein concentration in each lysate; an aliquot of each lysate was then diluted with sample buffer (50 mM Tris, 2% SDS, 0.1% bromophenol blue and 10% glycerol) to a final concentration of 1–2 μg/μL and was analyzed by SDS-PAGE and western blotting.

### Trans-well migration assay

Cells (3.0 × 10^4^) were plated in the upper chamber of BD BioCoat Control Culture Inserts (24-well plates, 8-μm pore size; Becton Dickinson, San Jose, CA). Serum-free culture medium was added to each upper chamber, and medium containing 5% FCS was added to the bottom chamber. Cells were incubated on the membranes for 12 h. After this 12-h incubation, four independent visual fields were examined via microscopic observation to count the number of cells that had moved to the bottom chamber.

### Matrigel invasion assay

A two-chamber invasion assay was used to assess cell invasion (24-well plates, 8-μm pore size membrane coated with a layer of Matrigel extracellular matrix proteins; Becton Dickinson). Cells (4.0 × 10^4^) suspended in serum-free medium were seeded into the upper chamber and allowed to invade towards a 5% FCS chemoattractant in the lower chamber. After a 20-h incubation, four independent visual fields were examined via microscopic observation to count the number of cells that had moved to the bottom chamber.

### Determination of patterns of protein phosphorylation

Relative protein phosphorylation levels of 38 selected proteins in scrambled control-siRNA transfected S2-013 cells and *CCDC88A*-siRNA transfected S2-013 cells were obtained by profiling 46 specific phosphorylation sites using the Proteome Profiler Human Phospho-Kinase Array Kit ARY003 from R&D Systems (Minneapolis, MN), according to the manufacturer’s instructions. Briefly, cells were rinsed with PBS, and 1 × 10^7^ cells/ml lysis buffer were solubilized with permanent shaking at 4 °C for 30 min. Aliquots of the lysates were stored frozen at −80 °C. Membranes with spotted catcher antibodies were incubated with diluted cell lysates at 4 °C overnight. Thereafter, cocktails of biotinylated detection antibodies were added at room temperature for 2 h. Phosphorylated proteins were detected using a horseradish peroxidase conjugated anti-mouse/rabbit antibody. Blots were then incubated with an enhanced chemiluminescence solution for 1 min for signal detection.

### Statistical analysis

The StatFlex software (Ver6; YUMIT, Osaka, Japan) and SAS software (Ver9.1.3; SAS Institute, Cary, NC) were used for statistical analysis. Student’s *t*-test was used for the comparison of continuous variables. Fisher’s exact test was used to assess the association between CCDC88A expression levels and clinicopathological parameters. The following parameters were examined: age, sex and the TNM classification or pathological stage using the Japan Pancreas Society [30] and the International Union against Cancer [31] score systems. Overall survival time was measured from the date of surgery to the date of death due to any cause or last clinical follow-up as determined by review of electronic medical records. Survival curves were plotted using the Kaplan-Meier method and were compared using the log-rank test (Mantel-Cox). Survival rates are given as median values and interquartile ranges (IQRs). ROC curves [14] were generated and analyzed using StatFlex software. Independent factors for overall survival including age, sex and pathological stage were assessed using Cox proportional hazards regression analysis. The relative hazard for each patient was calculated from coefficients determined by Cox regression. P values of < 0.05 were considered to be statistically significant. All tests were two-tailed.

## Additional files

Additional file 1: Figure S1. Roles of CCDC88A in the formation of cell protrusions in PANC-1 cells. a. Confocal Z stack images of PANC-1 cells that were transiently transfected with scrambled control-siRNA (Scr) or *CCDC88A*-siRNA (siCCDC88A). The transfected cells were incubated on fibronectin, and were subsequently stained with anti-CCDC88A antibody (green) and phalloidin (red). The lower and right panels in the confocal Z stack show a vertical cross-section (yellow lines) through the cells. Arrows, peripheral actin structures in cell protrusions of control-siRNA transfected cells. Blue, nuclear DAPI staining. Bars, 10 μm. b. Quantification of the data shown in Figure S1a. *Columns*, mean; *bars*, SD. **p* < 0.001 compared with Scr-transfected controls (Student’s *t*-test). c. Confocal immunofluorescence microscopic images of PANC-1 cells that had been transfected with *CCDC88A*-siRNA and were subsequently transfected with a myc-tagged CCDC88A-rescue construct. After 48 h, the cells were incubated on fibronectin. Cells were stained with anti-myc antibody (green), anti-CCDC88A antibody (red) and phalloidin (violet). Arrows, cell protrusions reproduced by myc-tagged CCDC88A in *CCDC88A*-siRNA transfected cells. Bars, 10 μm. d. Quantification of the data shown in Figure S1c; the values represent the number of cells with fibronectin-mediated cell protrusions in which peripheral actin structures were increased. All cells in four fields per group were scored. Data are derived from three independent experiments. *Columns*, mean; *bars*, SD. **p* < 0.001 compared with corresponding *CCDC88A*-siRNA transfected PANC-1 cells that were transfected with mock vector (Student’s *t*-test). e. Confocal Z stack images showing nuclear DAPI staining (blue) and the accumulation of myc-tagged CCDC88A (green) in fibronectin-stimulated CCDC88A-siRNA transfected PANC-1 cells transfected with the myc-tagged CCDC88A-rescue construct. Arrows, myc-tagged CCDC88A accumulated in cell protrusions. The lower and right panels of the confocal Z stack show a vertical cross-section (yellow lines) through the cells. Bar, 10 μm. (DOCX 1733 kb)
Additional file 2: Figure S2. Roles of CCDC88A in regulating the activation of Akt in PANC-1 cells. a. Scr or siCCDC88A oligos were transfected into PANC-1 cells. After 48 h, the cells were incubated on fibronectin for 5 h, and whole cell lysates were prepared for western blot analysis using anti-CCDC88A, anti-Akt and anti-phosphorylated Akt antibodies. b. Confocal immunofluorescence microscopic images of PANC-1 cells that were transiently transfected with scrambled control-siRNA or CCDC88A-siRNA. After 48 h, the cells were incubated on fibronectin for 5 h and were then stained with anti-phosphorylated Akt antibody (green), anti-CCDC88A antibody (red) and phalloidin (violet; actin filaments). Blue, nuclear DAPI staining. Bars, 10 μm. c. PANC-1 cells were pretreated with or without 5 μM of the Akt inhibitor triciribine for 2 h, following which the cells were plated on migration (left panel) and Matrigel invasion (right panel) chambers. Migrated cells in four fields per group were counted. Data are representative of three independent experiments. *Columns*, mean; *bars*, SD. d. PANC-1 cells were pretreated with or without 100 ng/mL IGF-1 for 10 min, and whole cell lysates were prepared for western blot analysis using anti-CCDC88A, anti-phosphorylated CCDC88A, anti-PI3K, anti-phosphorylated PI3K, anti-Akt, and anti-phosphorylated Akt antibodies. e. PANC-1 cells were pretreated with or without 100 ng/mL IGF-1 for 10 min and the cells were then plated on migration (left panel) and Matrigel invasion (right panel) chambers. Migrated cells in four fields per group were counted. Data are representative of three independent experiments. *Columns*, mean; *bars*, SD. f. Confocal immunofluorescence microscopic images of PANC-1 cells that were cultured on fibronectin with or without IGF-1 stimulation following which the cells were stained with anti-phosphorylated Akt antibody (green), anti-CCDC88A antibody (red) and phalloidin (violet; actin). Arrows, phosphorylated Akt accumulated in cell protrusions; arrowheads, CCDC88A accumulated in cell protrusions. Bars, 10 μm. (DOCX 1979 kb)
Additional file 3: Figure S3. Subcellular localization of CCDC88A and AMPK1 in PANC-1 cells. Confocal immunofluorescence microscopic images of PANC-1 cells that were cultured on fibronectin and were then labeled with anti-AMPK1 antibody (green), anti-CCDC88A antibody (red) and phalloidin (violet; actin filaments). Arrows, AMPK1 localized in cell protrusions. Blue, nuclear DAPI staining. Bar, 10 μm. (DOCX 631 kb)
