# Supplementary material for: CCDC88A, a prognostic factor for human pancreatic cancers, promotes the motility and invasiveness of pancreatic cancer cells
Source: J Exp Clin Cancer Res. 2016 Dec 5;35:190. doi: 10.1186/s13046-016-0466-0 (PMC5139074; doi:10.1186/s13046-016-0466-0)
Supplement: Additional file 1: Figure S1. — Roles of CCDC88A in the formation of cell protrusions in PANC-1 cells. a. Confocal Z stack images of PANC-1 cells that were transiently transfected with scrambled control-siRNA (Scr) or CCDC88A-siRNA (siCCDC88A). The transfected cells were incubated on fibronectin, and were subsequently stained with anti-CCDC88A antibody (green) and phalloidin (red). The lower and right panels in the confocal Z stack show a vertical cross-section (yellow lines) through the cells. Arrows, peripheral actin structures in cell protrusions of control-siRNA transfected cells. Blue, nuclear DAPI staining. Bars, 10 μm. b. Quantification of the data shown in Figure S1a. Columns, mean; bars, SD. *p < 0.001 compared with Scr-transfected controls (Student’s t-test). c. Confocal immunofluorescence microscopic images of PANC-1 cells that had been transfected with CCDC88A-siRNA and were subsequently transfected with a myc-tagged CCDC88A-rescue construct. After 48 h, the cells were incubated on fibronectin. Cells were stained with anti-myc antibody (green), anti-CCDC88A antibody (red) and phalloidin (violet). Arrows, cell protrusions reproduced by myc-tagged CCDC88A in CCDC88A-siRNA transfected cells. Bars, 10 μm. d. Quantification of the data shown in Figure S1c; the values represent the number of cells with fibronectin-mediated cell protrusions in which peripheral actin structures were increased. All cells in four fields per group were scored. Data are derived from three independent experiments. Columns, mean; bars, SD. *p < 0.001 compared with corresponding CCDC88A-siRNA transfected PANC-1 cells that were transfected with mock vector (Student’s t-test). e. Confocal Z stack images showing nuclear DAPI staining (blue) and the accumulation of myc-tagged CCDC88A (green) in fibronectin-stimulated CCDC88A-siRNA transfected PANC-1 cells transfected with the myc-tagged CCDC88A-rescue construct. Arrows, myc-tagged CCDC88A accumulated in cell protrusions. The lower and right panels of the confocal Z stack [file 13046_2016_466_MOESM1_ESM.docx]

**Roles of CCDC88A in the formation of cell protrusions in PANC-1 cells.**

a. Confocal Z stack images of PANC-1 cells that were transiently transfected with scrambled control-siRNA (Scr) or *CCDC88A*-siRNA (siCCDC88A). The transfected cells were incubated on fibronectin, and were subsequently stained with anti-CCDC88A antibody (green) and phalloidin (red). The lower and right panels in the confocal Z stack show a vertical cross-section (yellow lines) through the cells. Arrows, peripheral actin structures in cell protrusions of control-siRNA transfected cells. Blue, nuclear DAPI staining. Bars, 10 µm.

b. Quantification of the data shown in Figure S1a. *Columns*, mean; *bars*, SD. **p* < 0.001 compared with Scr-transfected controls (Student’s *t*-test).

c. Confocal immunofluorescence microscopic images of PANC-1 cells that had been transfected with *CCDC88A*-siRNA and were subsequently transfected with a myc-tagged CCDC88A-rescue construct. After 48 h, the cells were incubated on fibronectin. Cells were stained with anti-myc antibody (green), anti-CCDC88A antibody (red) and phalloidin (violet). Arrows, cell protrusions reproduced by myc-tagged CCDC88A in *CCDC88A*-siRNA transfected cells. Bars, 10 µm.

d. Quantification of the data shown in Figure S1c; the values represent the number of cells with fibronectin-mediated cell protrusions in which peripheral actin structures were increased. All cells in four fields per group were scored. Data are derived from three independent experiments. *Columns*, mean; *bars*, SD. **p* < 0.001 compared with corresponding *CCDC88A*-siRNA transfected PANC-1 cells that were transfected with mock vector (Student’s *t*-test).

e. Confocal Z stack images showing nuclear DAPI staining (blue) and the accumulation of myc-tagged CCDC88A (green) in fibronectin-stimulated CCDC88A-siRNA transfected PANC-1 cells transfected with the myc-tagged CCDC88A-rescue construct. Arrows, myc-tagged CCDC88A accumulated in cell protrusions. The lower and right panels of the confocal Z stack show a vertical cross-section (yellow lines) through the cells. Bar, 10 µm.
