# Supplementary material for: CCDC88A, a prognostic factor for human pancreatic cancers, promotes the motility and invasiveness of pancreatic cancer cells
Source: J Exp Clin Cancer Res. 2016 Dec 5;35:190. doi: 10.1186/s13046-016-0466-0 (PMC5139074; doi:10.1186/s13046-016-0466-0)
Supplement: Additional file 2: Figure S2. — Roles of CCDC88A in regulating the activation of Akt in PANC-1 cells. a. Scr or siCCDC88A oligos were transfected into PANC-1 cells. After 48 h, the cells were incubated on fibronectin for 5 h, and whole cell lysates were prepared for western blot analysis using anti-CCDC88A, anti-Akt and anti-phosphorylated Akt antibodies. b. Confocal immunofluorescence microscopic images of PANC-1 cells that were transiently transfected with scrambled control-siRNA or CCDC88A-siRNA. After 48 h, the cells were incubated on fibronectin for 5 h and were then stained with anti-phosphorylated Akt antibody (green), anti-CCDC88A antibody (red) and phalloidin (violet; actin filaments). Blue, nuclear DAPI staining. Bars, 10 μm. c. PANC-1 cells were pretreated with or without 5 μM of the Akt inhibitor triciribine for 2 h, following which the cells were plated on migration (left panel) and Matrigel invasion (right panel) chambers. Migrated cells in four fields per group were counted. Data are representative of three independent experiments. Columns, mean; bars, SD. d. PANC-1 cells were pretreated with or without 100 ng/mL IGF-1 for 10 min, and whole cell lysates were prepared for western blot analysis using anti-CCDC88A, anti-phosphorylated CCDC88A, anti-PI3K, anti-phosphorylated PI3K, anti-Akt, and anti-phosphorylated Akt antibodies. e. PANC-1 cells were pretreated with or without 100 ng/mL IGF-1 for 10 min and the cells were then plated on migration (left panel) and Matrigel invasion (right panel) chambers. Migrated cells in four fields per group were counted. Data are representative of three independent experiments. Columns, mean; bars, SD. f. Confocal immunofluorescence microscopic images of PANC-1 cells that were cultured on fibronectin with or without IGF-1 stimulation following which the cells were stained with anti-phosphorylated Akt antibody (green), anti-CCDC88A antibody (red) and phalloidin (violet; actin). Arrows, phosphorylated Akt accumulated in cell protrusions; arrowheads, CC [file 13046_2016_466_MOESM2_ESM.docx]

**Roles of CCDC88A in regulating the activation of Akt in PANC-1 cells.**

a. Scr or siCCDC88A oligos were transfected into PANC-1 and PANC-1 cells. After 48 h, the cells were incubated on fibronectin for 5 h, and whole cell lysates were prepared for western blot analysis using anti-CCDC88A, anti-Akt and anti-phosphorylated Akt antibodies. Data are representative of three independent experiments.

b. Confocal immunofluorescence microscopic images of PANC-1 cells that were transiently transfected with scrambled control-siRNA or *CCDC88A*-siRNA. After 48 h, the cells were incubated on fibronectin for 5 h and were then stained with anti-phosphorylated Akt antibody (green), anti-CCDC88A antibody (red) and phalloidin (violet; actin filaments). Blue, nuclear DAPI staining. Bars, 10 µm.

c. PANC-1 cells were pretreated with or without 5 μM of the Akt inhibitor triciribine for 2 h, following which the cells were plated on motility (left panel) and Matrigel invasion (right panel) chambers. Migrated cells in four fields per group were counted. Data are representative of three independent experiments. *Columns*, mean; *bars*, SD.

d. PANC-1 cells were pretreated with or without 100 ng/mL IGF-1 for 10 min, and whole cell lysates were prepared for western blot analysis using anti-CCDC88A, anti-phosphorylated CCDC88A, anti-PI3K, anti-phosphorylated PI3K, anti-Akt, and anti-phosphorylated Akt antibodies. Data are representative of three independent experiments.

e. PANC-1 cells were pretreated with or without 100 ng/mL IGF-1 for 10 min and the cells were then plated on motility (left panel) and Matrigel invasion (right panel) chambers. Migrated cells in four fields per group were counted. Data are representative of three independent experiments. *Columns*, mean; *bars*, SD.

f. Confocal immunofluorescence microscopic images of PANC-1 cells that were cultured on fibronectin with or without IGF-1 stimulation following which the cells were stained with anti-phosphorylated Akt antibody (green), anti-CCDC88A antibody (red) and phalloidin (violet; actin). Arrows, phosphorylated Akt accumulated in cell protrusions; arrowheads, CCDC88A accumulated in cell protrusions. Blue, nuclear DAPI staining. Bars, 10 µm.
