# Supplementary material for: CCDC88A, a prognostic factor for human pancreatic cancers, promotes the motility and invasiveness of pancreatic cancer cells
Source: J Exp Clin Cancer Res. 2016 Dec 5;35:190. doi: 10.1186/s13046-016-0466-0 (PMC5139074; doi:10.1186/s13046-016-0466-0)
Supplement: Additional file 3: Figure S3. — Subcellular localization of CCDC88A and AMPK1 in PANC-1 cells. Confocal immunofluorescence microscopic images of PANC-1 cells that were cultured on fibronectin and were then labeled with anti-AMPK1 antibody (green), anti-CCDC88A antibody (red) and phalloidin (violet; actin filaments). Arrows, AMPK1 localized in cell protrusions. Blue, nuclear DAPI staining. Bar, 10 μm. (DOCX 631 kb) [file 13046_2016_466_MOESM3_ESM.docx]

**Subcellular localization of CCDC88A and AMPK1 in PANC-1 cells.**

Confocal immunofluorescence microscopic images of PANC-1 cells that were cultured on fibronectin and were then labeled with anti-AMPK1 antibody (green), anti-CCDC88A antibody (red) and phalloidin (violet; actin filaments). Arrows, AMPK1 localized in cell protrusions. Blue, nuclear DAPI staining. Bar, 10 μm.
